# Factors associated with take-home naloxone kit usage in British Columbia: an analysis of administrative data

**DOI:** 10.1186/s13011-022-00452-8

**Published:** 2022-03-31

**Authors:** Victor Lei, Max Ferguson, Rachael Geiger, Sierra Williams, Lisa Liu, Jane A. Buxton

**Affiliations:** 1 School of Population and Public Health, University of British Columbia, Vancouver, BC Canada; 2 BC Centre for Disease Control, Vancouver, BC Canada

**Keywords:** Naloxone, Take-home naloxone, Opioid antagonist, Overdose, Toxicity, Harm reduction

## Abstract

**Background:**

The British Columbia (BC) Take-Home Naloxone (THN) program provides naloxone to people at risk of experiencing or witnessing an opioid overdose for use in reversing suspected overdose events. This study seeks to examine trends and correlates of individuals obtaining a THN kit in BC between 2017 and 2020.

**Methods:**

Records of THN kits distributed between 2017 and 2020 were the primary source of data for this analysis. Frequency tables were used to describe characteristics of people obtaining kits from THN sites. Correlates of individuals obtaining a THN kit to replace a previous kit reported as used to reverse an overdose were assessed with multivariate logistic regression.

**Results:**

Between January 1, 2017, and December 31, 2020, 240,606 THN kits were reported distributed by registered sites to members of the public, with 90,011 records indicating that a kit was obtained to replace a previous kit that had been used to reverse an overdose. There was a significant trend in increasing kits reported used by year (*p* < 0.01). The kit recipient’s risk of overdose was a significant predictor of having reported using a THN kit, and the strength of the association was dependent on gender (Male: Adjusted odds ratio (AOR) 5.37 [95% confidence interval (CI) 5.08 – 5.67]; Female: AOR 8.35 [95% CI 7.90 – 8.82]; Trans and gender expansive: AOR 3.68 [95% CI 2.82 – 4.79]).

**Conclusions:**

Between 2017 and 2020, THN kits were used to reverse tens of thousands of overdose events in BC, with people at risk of overdose (i.e. people who use drugs [PWUD]) having greater odds of using a kit to reverse an overdose than those not at risk. Thus, PWUD are responsible for reversing the vast majority of overdoses. THN kits are being distributed to the people who use them most. However, additional strategies in conjunction with community-based naloxone distribution programs are needed to address the rising number of illicit drug toxicity deaths.

**Supplementary Information:**

The online version contains supplementary material available at 10.1186/s13011-022-00452-8.

## Background

Opioids are a class of drugs, either naturally derived from the poppy plant or produced synthetically, that are commonly used as painkillers [1]. Opioids bind to receptors in the central nervous system which, in addition to providing analgesic effects, can cause respiratory depression [2]. Opioid-mediated respiratory depression can lead to anoxic brain injury, coma, and death [3].

Rising mortality rates related to illicit drugs have been observed in the UK, parts of Africa, South-East Asia, and North America; the majority of which are attributable to opioids [4]. North America has been severely affected by the evolving drug toxicity crisis linked closely to synthetic opioids including fentanyl and its analogues. Almost 50,000 people died from overdose in the United States in 2019 [4], while over 19,000 deaths were caused by an opioid overdose between January 2016 and September 2020 in Canada [5]. In April 2016, the British Columbia (BC) Provincial Health Office declared a public health emergency in response to escalating numbers of opioid overdose-related deaths, driven by the emergence of fentanyl and its analogues in the illicit drug supply [6]. Since that announcement, more than 7,000 deaths have occurred as a result of illicit drug toxicity in BC [7].

Fentanyl is up to 100 times more potent than morphine and 20–40 times more potent than heroin, increasing the risk of accidental overdose [8]. Fentanyl also has a quicker onset than other opioids and is highly fat soluble, which enables rapid diffusion through the blood–brain barrier, further increasing toxicity [9].

Naloxone is a µ-opioid receptor antagonist that displaces opioids such as fentanyl and temporarily reverses their toxic effects [10]. It has been shown that naloxone has an onset of action within minutes, whether it be through intravenous, intramuscular, or intranasal routes of administration [11, 12]. Take-Home Naloxone (THN) programs have been implemented in various parts of the world, including BC, where the THN program has been in operation since 2012 [13]. These programs aim to make naloxone available within communities for bystander administration in the event of suspected overdose events [14].

BC’s provincial THN program was established as one of the first of its kind in Canada [15]. It was designed to provide low-barrier access to injectable naloxone and overdose response training for people who use drugs (PWUD) in areas with the highest rates of opioid overdose. Kits were made available at no charge to eligible participants through harm reduction sites, peer-led organizations, non-governmental organizations, hospitals, health units, emergency departments, supportive housing, and upon release from corrections facilities [16–18]. Removal of naloxone from the Prescription Drug List [19] enabled expanded access. In December 2016, BC’S THN program extended eligibility for kits to people who were not personally at risk of an overdose but were likely to witness one, such as friends and family members of PWUD [17, 19]. In December 2017, the BC THN program was rolled out in community pharmacies, also at no charge, greatly increasing access to kits, especially in remote regions [20]. Prior research indicates that people who are not personally at risk of overdose are more likely to obtain kits from pharmacy sites, especially when collecting a naloxone kit for the first time [21].

Evidence has shown that THN programs are effective at preventing opioid-overdose deaths in BC as well as in other countries around the world [22, 23]. In BC, between 2012 and 2018, where the outcome of a suspected opioid overdose was known, 98.1% of people who were administered naloxone survived their overdose [24]. Mathematical modelling estimates that 1,650 death events were averted in BC as a result of the THN program in the 20 months between April 2016 and December 2017 [25].

It is well established that community-based naloxone administration saves lives. However, less is known about factors associated with people using their kit. Therefore, this study aims to evaluate correlates of individuals obtaining a THN kit in BC between 2017 and 2020.

## Methods

### Data sources and study variables

BC’s provincial THN program is overseen by the British Columbia Centre for Disease Control (BCCDC) and supported by the BC Ministry of Health [13]. Registered distribution sites order THN kits, which are shipped from the BCCDC, and sites are asked to complete “distribution records” whenever kits are provided to the public (see Additional file 1). Characteristics of individuals receiving naloxone kits are collected in distribution records at the time an individual obtains a kit [26]. Sites are asked to return completed distribution records to the BCCDC on a monthly basis for data entry. Return of distribution records is not 100% complete, thus the number of kits reported distributed does not match the total number of kits that have been shipped.

Distribution records were the main source of data used in this study. Those distributing kits report the recipient’s gender (male, female, or other [trans and gender expansive]), whether they were personally at risk of overdose (yes or no), their age group (< 19, 19–30, 31–60, or > 60), and whether they were collecting a THN kit for the first time or as a replacement for a previous kit. For replacements, those distributing kits reported whether the recipient’s previous kit had been used or needed replacing for another reason, such as the kit being stolen, lost, confiscated, or the naloxone in the kit having expired, as the shelf life of naloxone is approximately two years.

Information provided by the distribution site includes the date of kit distribution and site-specific information such as unique site name, site ID, and city. The site-specific information is used to identify the geographic health region (Fraser Health, Interior Health, Island Health, Northern Health, or Vancouver Coastal Health) where each kit was distributed.

### Sample selection

Eligibility for the THN program was expanded in December 2016 to allow distribution to individuals that do not personally identify as at risk of an overdose, but were likely to witness and respond to an overdose. At this time, distribution records were updated to include a question about self-reported risk of overdose (i.e. whether someone is a PWUD). Because information about overdose risk was collected beginning in December 2016, we included only records from complete years after 2016 (2017–2020) in the study.

### Data analysis

We categorized THN kits that were reported to have been replaced for any reason other than use, as well as first kit distributions, as “Not Used” and compared to kits that were “Used.” A Cochran-Armitage test for trend was used to evaluate differences in the number of kits reported used over time. We calculated frequency distributions to describe characteristics of people replacing used kits and collecting kits for other reasons, people at risk and not at risk of overdose, and people who made up the three separate gender categories. The purposeful selection model-building technique described by Bursac et al. [27] was employed. We performed bivariate analyses to determine predictors for someone having reported using a kit, and associations of *p* < 0.25 were included in a multivariate logistic regression model. The final model was selected through a backwards selection approach based on the lowest Akaike’s information criteria (AIC) value [28]. Collinearity between variables was assessed by removing each variable from the final multivariate model and assessing changes to the standard error value of the main association of interest (risk of overdose and having reported using a kit). The absence of collinearity was confirmed by calculating variance inflation factor (VIF) values for all variables in the model. We used a likelihood ratio test (LRT) to determine the presence of effect modification by suspected variables on the main association of interest. All analyses were conducted using R 4.1.0 and R Studio Version 1.4.1717.

## Results

**Fig. 1:**
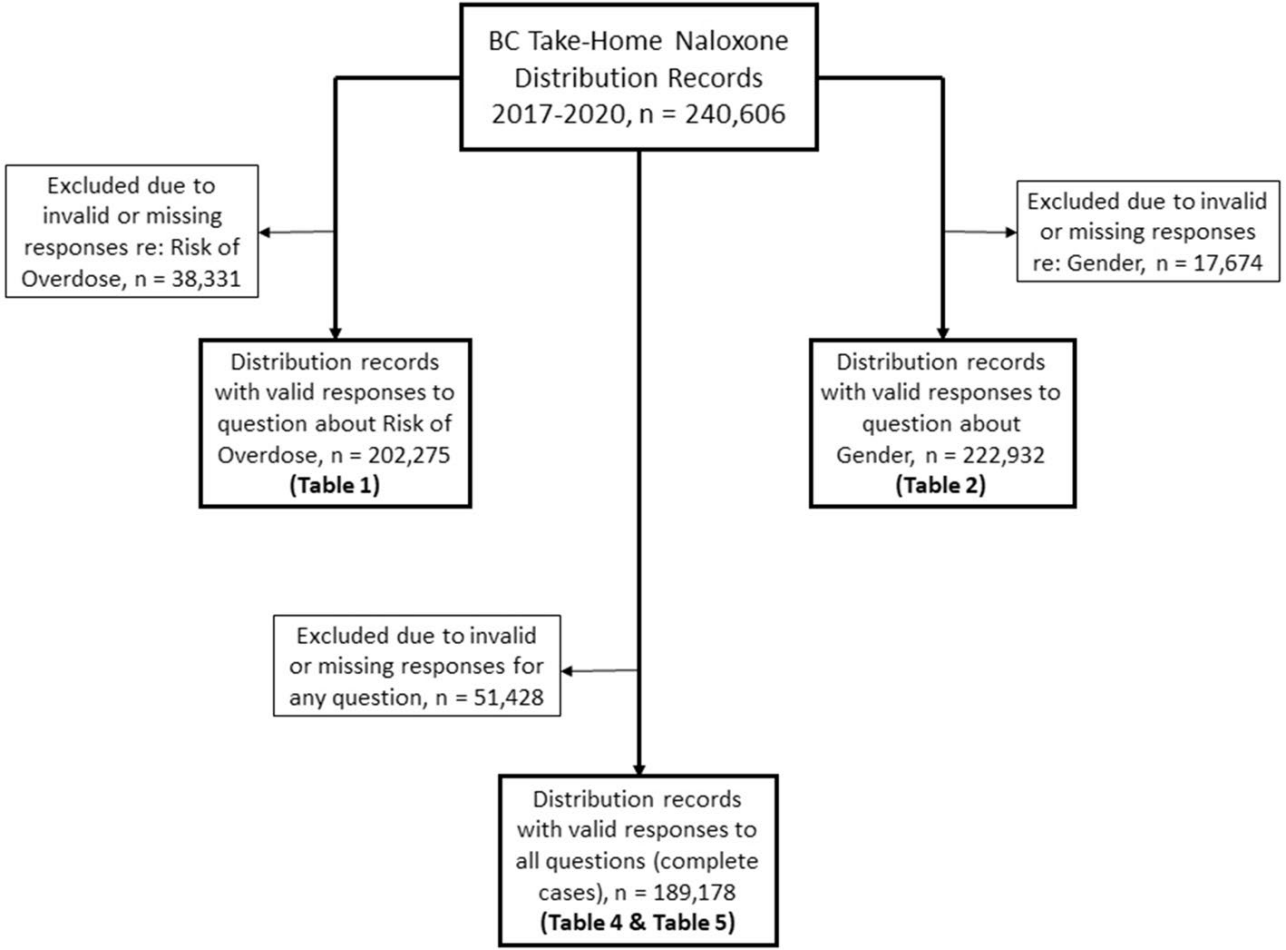
Study samples for sub-analyses from BC Take-Home Naloxone distribution records from January 1, 2017 to December 31, 2020

A total of 275,632 THN kits have been reported distributed since the inception of the BC THN program in 2012 through May 31, 2021 [29]. Of these, 240,606 records of kits distributed between January 1, 2017 and December 31, 2020 were included in the analysis. Figure 1 shows the number of records meeting inclusion criteria for each sub-analysis. The proportion of records with missing data for each variable are as follows: year: 0%, health region: 0.2%, risk of overdose: 15.9%, gender: 7.3%, age group: 8.4%. We used a complete case analysis approach, with 189,178 out of 240,606 (78.6%) distribution records used in the multivariable analysis.

### Overdose risk

Table 1 summarizes characteristics of BC THN kit recipients between 2017 and 2020, stratified by risk of overdose. Of the 240,606 distribution records received, 202,275 contained valid responses to the question about overdose risk and were included in Table 1 (Fig. 1). All variables with missing entries are classified as “unknown”.

**Table 1:** Summary characteristics of BC Take-Home Naloxone distribution data from January 1, 2017 to December 31, 2020, stratified by risk of overdose

Characteristics	Not Personally at Risk of Overdose (*N* = 51,641)	Personally at Risk of Overdose (*N* = 150,634)	Total (*N* = 202,275)	*p*-value of Chi Square Analysis
	**n (%)**	**n (%)**	n (%*)	
**Year**				< 0.001
2017	10,224 (30.4%)	23,426 (69.6%)	33,650 (16.6%)	
2018	18,487 (31.9%)	39,521 (68.1%)	58,008 (28.7%)	
2019	12,990 (25.7%)	37,633 (74.3%)	50,623 (25.0%)	
2020	9,940 (16.6%)	50,054 (83.4%)	59,994 (29.7%)	
**Health Region**				< 0.001
Fraser	10,486 (16.8%)	51,977 (83.2%)	62,463 (30.9%)	
Interior	13,712 (27.8%)	35,572 (72.2%)	49,284 (24.4%)	
Island	14,169 (30.8%)	31,900 (69.2%)	46,069 (22.8%)	
Northern	3,724 (22.1%)	13,143 (77.9%)	16,867 (8.3%)	
Vancouver Coastal	9,374 (34.5%)	17,777 (65.5%)	27,151 (13.4%)	
Unknown	176 (39.9%)	265 (60.1%)	441 (0.2%)	
**Kit Collection Reason**				< 0.001
Used	6,949 (8.4%)	75,667 (91.6%)	82,616 (40.8%)	
Not Used^a^	44,692 (37.3%)	74,967 (62.7%)	119,659 (59.2%)	
**Gender**				< 0.001
Male	19,446 (19.2%)	82,088 (80.8%)	101,534 (50.2%)	
Female	28,209 (32.0%)	60,054 (68.0%)	88,263 (43.6%)	
Trans and Gender Expansive	2,081 (43.7%)	2,679 (56.3%)	4,760 (2.4%)	
Unknown	1,905 (24.7%)	5,813 (75.3%)	7,718 (3.8%)	
**Age Group**				< 0.001
Under 19	2,589 (26.4%)	7,203 (73.6%)	9,792 (4.8%)	
19—30	15,991 (24.0%)	50,555 (76.0%)	66,546 (32.9%)	
31—60	27,392 (24.9%)	82,515 (75.1%)	109,907 (54.3%)	
Over 60	2,733 (44.1%)	3,463 (55.9%)	6,196 (3.1%)	
Unknown	2,936 (29.9%)	6,898 (70.1%)	9,834 (4.9%)	

^*^Column percentages

^a^Take-home naloxone kit was recipient’s first kit or replacement due to a previous kit being lost, stolen, confiscated or expired

As shown in Table 1, the number and proportion of people collecting THN kits who were personally at risk of overdose increased, from 69.6% in 2017 to 83.4% in 2020. The vast majority (91.6%) of kits reported used were by people who were at risk of overdose. The proportion of people who received a kit and were at risk of overdose was more than 70% for all age groups except those over 60, where it was 55.9%.

### Gender

Table 2 presents summary characteristics of BC THN kit recipients between 2017 and 2020, stratified by gender. Of the 240,606 distribution records received, 222,932 contained valid responses to the question about gender and were included in Table 2 (Fig. 1). Men collected more kits (48.4% of all kits) than women (42.1% of all kits), and men (42.2%) were more likely to report replacing a kit because it had been used than women (36.3%) or trans and gender expansive people (30.8%). Men receiving a kit were also more likely to report being at risk of overdose (80.8%) than women (68.0%) or trans and gender expansive individuals (56.3%).

**Table 2:** Summary characteristics of BC Take-Home Naloxone distribution data from January 1, 2017 to December 31, 2020, stratified by gender

Characteristics	Male (*N* = 116,423)	Female (*N* = 101,237)	Trans and Gender Expansive (*N* = 5,272)	Total (*N* = 222,932)	*p*-value of Chi Square Analysis
	**n (%)**	**n (%)**	**n (%)**	n (%*)	
**Year**					< 0.001
2017	30,966 (54.2%)	25,497 (44.6%)	692 (1.2%)	57,155 (25.6%)	
2018	30,229 (52.8%)	25,882 (45.2%)	1,133 (2.0%)	57,244 (25.7%)	
2019	24,663 (49.2%)	24,606 (49.1%)	859 (1.7%)	50,128 (22.5%)	
2020	30,565 (52.3%)	25,252 (43.2%)	2,588 (4.4%)	58,405 (26.2%)	
**Health Region**					< 0.001
Fraser	36,704 (54.6%)	29,617 (44.1%)	846 (1.3%)	67,167 (30.1%)	
Interior	26,459 (49.8%)	24,992 (47.1%)	1,634 (3.1%)	53,085 (23.8%)	
Island	26,518 (51.4%)	23,334 (45.2%)	1,743 (3.4%)	51,595 (23.1%)	
Northern	8,433 (47.8%)	8,985 (50.9%)	228 (1.3%)	17,646 (7.9%)	
Vancouver Coastal	18,090 (54.8%)	14,084 (42.7%)	818 (2.5%)	32,992 (14.8%)	
Unknown	219 (49.0%)	225 (50.3%)	3 (0.7%)	447 (0.2%)	
**Kit Collection Reason**					< 0.001
Not Used^a^	67,253 (49.7%)	64,525 (47.6%)	3,647 (2.7%)	135,425 (60.7%)	
Used	49,170 (56.2%)	36,712 (42.0%)	1,625 (1.9%)	87,507 (39.3%)	
**Age Group**					< 0.001
Under 19	4,704 (46.0%)	5,216 (51.0%)	309 (3.0%)	10,229 (4.6%)	
19—30	39,079 (52.0%)	34,876 (46.4%)	1,137 (1.5%)	75,092 (33.7%)	
31—60	66,323 (53.5%)	56,130 (45.3%)	1,494 (1.2%)	123,947 (55.6%)	
Over 60	3,950 (55.5%)	3,039 (42.7%)	122 (1.7%)	7,111 (3.2%)	
Unknown	2,367 (36.1%)	1,976 (30.2%)	2,210 (33.7%)	6,553 (2.9%)	
**Risk of Overdose**					< 0.001
Not at Risk	19,446 (39.1%)	28,209 (56.7%)	2,081 (4.2%)	49,736 (22.3%)	
At Risk	82,088 (56.7%)	60,054 (41.5%)	2,679 (1.8%)	144,821 (65.0%)	
Unknown	14,889 (52.5%)	12,974 (45.7%)	512 (1.8%)	28,375 (12.7%)	

^*^Column percentages

^a^Take-home naloxone kit was recipient’s first kit or replacement due to a previous kit being lost, stolen, confiscated or expired

### Correlates of individuals having reported using a THN kit

Table 3 summarizes characteristics of THN kit recipients between 2017 and 2020, stratified by whether a kit was reported as used, as well as bivariate analytic results (unadjusted odds ratios and 99% confidence intervals).

**Table 3:** Unadjusted odds ratios and 99% confidence intervals for odds of reporting using a kit vs. not reporting using a kit

Characteristics	Kit Used (Replacement—Used) (*N* = 90,011)	Kit Not Used^a^ (1st Kit or Replacement-Other) (*N* = 150,595)	Total (*N* = 240,606)	Bivariate Analysis	
				**UOR (99% CI)**	***p*-value**
	**n (%)**	**n (%)**	n (%*)		
**Year**					
2017	15,523 (24.5%)	47,824 (75.5%)	63,347 (26.3%)	1.00	-
2018	21,309 (35.1%)	39,370 (64.9%)	60,679 (25.2%)	1.48 (1.42—1.54)	< 0.001
2019	21,009 (39.9%)	31,691 (60.1%)	52,700 (21.9%)	1.83 (1.75—1.90)	< 0.001
2020	32,170 (50.4%)	31,710 (49.6%)	63,880 (26.5%)	3.04 (2.92—3.16)	< 0.001
**Health Region**					
Fraser	28,392 (39.3%)	43,827 (60.7%)	72,219 (30.0%)	1.00	-
Interior	23,273 (40.2%)	34,664 (59.8%)	57,937 (24.1%)	1.11 (1.08 – 1.15)	< 0.001
Island	20,806 (36.8%)	35,657 (63.2%)	56,463 (23.5%)	0.96 (0.93 – 0.99)	0.001
Northern	6,129 (33.1%)	12,398 (66.9%)	18,527 (7.7%)	0.72 (0.69 – 0.76)	< 0.001
Vancouver Coastal	11,385 (32.5%)	23,601 (67.5%)	34,986 (14.5%)	0.70 (0.67 – 0.73)	< 0.001
Unknown	26 (5.5%)	448 (94.5%)	474 (0.2%)	-	-
**Risk of Overdose**					
Not at Risk	6,949 (13.5%)	44,692 (86.5%)	51,641 (21.5%)	1.00	-
At Risk	75,667 (50.2%)	74,967 (49.8%)	150,634 (62.6%)	7.02 (6.76—7.29)	< 0.001
Unknown	7,395 (19.3%)	30,936 (80.7%)	38,331 (15.9%)	-	-
**Gender**					
Male	49,170 (42.2%)	67,253 (57.8%)	116,423 (48.4%)	1.00	-
Female	36,712 (36.3%)	64,525 (63.7%)	101,237 (42.1%)	0.79 (0.77—0.81)	< 0.001
Trans or Gender Expansive	1,625 (30.8%)	3,647 (69.2%)	5,272 (2.2%)	0.48 (0.43—0.53)	< 0.001
Unknown	2,504 (14.2%)	15,170 (85.8%)	17,674 (7.3%)	-	-
**Age Group**					
Under 19	2,772 (25.6%)	8,071 (74.4%)	10,843 (4.5%)	1.00	-
19—30	29,710 (38.9%)	46,599 (61.1%)	76,309 (31.7%)	1.82 (1.71—1.93)	< 0.001
31—60	52,545 (41.7%)	73,325 (58.3%)	125,870 (52.3%)	2.04 (1.92—2.17)	< 0.001
Over 60	1,887 (26.0%)	5,380 (74.0%)	7267 (3.0%)	0.99 (0.90—1.09)	0.834
Unknown	3,097 (15.2%)	17,220 (84.8%)	20,317 (8.4%)	-	-

Abbreviations: *UOR* unadjusted odds ratio, *CI* confidence interval

^*^Column percentages

^a^Take-home naloxone kit was recipient’s first kit or replacement due to a previous kit being lost, stolen, confiscated or expired

In total, 240,606 distribution records were entered, with 90,011 (37.4%) of kits collected reported as replacements for used kits. The number of kits reported to have been used increased from 15,523 (24.5% of all kits distributed) in 2017 to 32,170 (50.4% of all kits distributed) in 2020. Despite fewer total kits reported distributed in 2018 (60,679) and 2019 (52,700) compared with 2017 (63,347) and 2020 (63,880), the proportion of kits that were reported as used increased every year. Meanwhile, the proportion of kits reported distributed for reasons other than to replace a used kit (first kit received by an individual, or previous kit was lost, stolen, confiscated, or expired) has decreased over time. This is especially true for first kits, with the number of kits being collected by individuals for the first time declining from 36,886 (58.2% of all kits distributed) in 2017 to 20,887 (32.7% of all kits distributed) in 2020 (see Additional file 2).

When picking up a THN kit, people who identified as being at risk of overdose reported replacing a previously used kit 50.2% of the time, while individuals not at risk of overdose reported replacing a previously used kit only 13.5% of the time. Northern Health, the health region with the second-highest likelihood of someone being at risk of overdose (77.9%) saw the second-lowest rate of kit use (33.1%).

There was a strong association between self-reported risk of overdose and THN kit use. Bivariate analysis of this relationship showed that people who were at risk of overdose had more than seven times the odds (UOR: 7.02, 99% CI: 6.76 – 7.29) of having reported using a kit than those not personally at risk of overdose.

We explored potential effect modification by gender on the association between risk of overdose and having used a naloxone kit. We found that the strength of the relationship between risk of overdose and having used a kit depended on gender with a significant gender-risk of overdose effect modification term (LRT *p* < 0.01). Table 4 presents the joint effect of the two risk factors (risk of overdose and gender), unstratified and stratified by gender, on having reported using a kit. Females at risk of overdose had 8.35 times the odds (99% CI: 7.90 – 8.82) of having reported using a kit than females not at risk of overdose, after adjusting for year, health region, and age group. Males at risk of overdose had 5.37 times the odds (99% CI: 5.08 – 5.67) of having reported using a kit than males not at risk of overdose, and trans and gender-expansive people had 3.68 times the odds (99% CI: 2.82 – 4.79) of having reported using a kit than someone of the same population that was not at risk of overdose, after adjusting for the same. For all three gender strata, the odds that someone reported using a kit increased each year from 2017 to 2020, with the odds more than doubling (men: AOR 2.77 [99% CI 2.63 – 2.93], women: AOR 2.83 [99% CI 2.65 – 3.02], trans and gender-diverse: AOR 2.20 [99% CI 1.41 – 3.43]) in 2020 compared with 2017. There is a significant trend of increasing kit use by year (Cochran-Armitage test for trend p-value < 0.01 for all strata; see Additional file 3 for kits reported used by year, stratified by gender).

**Table 4:** Effect modification by gender on association between overdose risk and having reported using a kit – Adjusted odds ratios and 99% confidence intervals for odds of having reported using a kit vs not having reported using a kit, stratified by gender

Variable	Unstratified (*N* = 189,178)		Gender: Male (*N* = 99,738)		Gender: Female (*N* = 86,633)		Gender: Other^A^ (*N* = 2,807)	
	AOR* (99% CI)	***p*-value**	**AOR (99% CI)**	***p*-value**	**AOR (99% CI)**	***p*-value**	**AOR (99% CI)**	***p*-value**
**Risk of Overdose**								
Not at Risk	1.00	-	1.00	-	1.00	-	1.00	-
At Risk	6.82 (6.57 – 7.09)	< 0.001	5.37 (5.08 – 5.67)	< 0.001	8.35 (7.90 – 8.82)	< 0.001	3.68 (2.82 – 4.79)	< 0.001
**Year**								
2017	1.00	-	1.00	-	1.00	-	1.00	-
2018	1.59 (1.53 – 1.66)	< 0.001	1.59 (1.50 – 1.68)	< 0.001	1.63 (1.52 – 1.74)	< 0.001	1.25 (0.79 – 1.97)	0.206
2019	1.85 (1.77 – 1.93)	< 0.001	1.85 (1.75 – 1.96)	< 0.001	1.87 (1.75 – 2.00)	< 0.001	1.17 (0.72 – 1.90)	0.396
2020	2.76 (2.65 – 2.88)	< 0.001	2.77 (2.63 – 2.93)	< 0.001	2.83 (2.65 – 3.02)	< 0.001	2.20 (1.41 – 3.43)	< 0.001
**Health Region**								
Fraser	1.00	-	1.00	-	1.00	-	1.00	-
Interior	1.35 (1.30 – 1.40)	< 0.001	1.46 (1.39 – 1.53)	< 0.001	1.27 (1.20 – 1.34)	< 0.001	0.50 (0.35 – 0.72)	< 0.001
Island	1.35 (1.30 – 1.40)	< 0.001	1.49 (1.42 – 1.56)	< 0.001	1.20 (1.14 – 1.27)	< 0.001	1.11 (0.81 – 1.53)	0.388
Northern	0.70 (0.67 – 0.74)	< 0.001	0.74 (0.69 – 0.79)	< 0.001	0.66 (0.61 – 0.71)	< 0.001	1.06 (0.61 – 1.84)	0.792
Vancouver Coastal	0.99 (0.95 – 1.04)	0.664	1.07 (1.01 – 1.14)	0.001	0.91 (0.85 – 0.97)	< 0.001	0.76 (0.53 – 1.10)	0.059
**Age Group**								
Under 19	1.00	-	1.00	-	1.00	-	1.00	-
19—30	1.94 (1.81 – 2.07)	< 0.001	1.84 (1.67 – 2.03)	< 0.001	2.05 (1.86 – 2.25)	< 0.001	1.87 (1.21 – 2.89)	< 0.001
31—60	2.19 (2.05 – 2.34)	< 0.001	2.14 (1.95 – 2.35)	< 0.001	2.24 (2.04 – 2.46)	< 0.001	1.83 (1.20 – 2.80)	< 0.001
Over 60	1.35 (1.22 – 1.49)	< 0.001	1.36 (1.18 – 1.56)	< 0.001	1.29 (1.10 – 1.52)	< 0.001	1.35 (0.66 – 2.77)	0.276

Abbreviations: *AOR* adjusted odds ratio

^*^ Odds ratio between each group and the reference group for each variable, after adjusting for all other variables: overdose risk, year, health region, age group

In all strata, the youngest age group (individuals < 19 years old) had the lowest odds of using a kit. Adults between 19–30 years old (men: AOR 1.84 [99% CI 1.67 – 2.03], women: AOR: 2.05 [99% CI 1.86 – 2.25], trans and gender diverse individuals: AOR 1.87 [99% CI 1.21 – 2.89]) and between 31–60 years old (men: AOR 2.14 [99% CI 1.95 – 2.35], women: AOR: 2.24 [99% CI 2.04 – 2.46], trans and gender diverse individuals: AOR 1.83 [99% CI 1.20 – 2.80]) had approximately twice the odds of having reported using a kit than people under 19.

We ruled out collinearity between variables by confirming equivalent standard errors between models that included and excluded each variable, as well as by assessing VIFs.

Table 5 shows the bivariate frequency distributions by overdose risk and having reported using a kit, stratified by gender.

**Table 5:** Bivariate frequency distributions between overdose risk and kits used, stratified by gender

	**Kit Used**	**Kit Not Used**
	**n (%)**	**n (%)**
**Unstratified**		
At Risk	72,807 (51.5%)	68,543 (48.5%)
Not at Risk	6,287 (13.1%)	41,551 (86.9%)
**Male**		
At Risk	41,487 (51.4%)	39,171 (48.6%)
Not at Risk	3,066 (16.1%)	16,014 (83.9%)
**Female**		
At Risk	30,681 (52.0%)	28,306 (48.0%)
Not at Risk	3,070 (11.1%)	24,576 (88.9%)
**Trans and Gender Expansive**		
At Risk	629 (37.1%)	1,066 (62.9%)
Not at Risk	151 (13.6%)	961 (86.4%)

## Discussion

This study provides an overview of BC THN kit distribution during the years of 2017 to 2020. The proportion of THN kits reported to have been used to reverse an overdose increased in each subsequent year, from 24.5% in 2017 to 50.4% in 2020. After reaching a peak in 2018, the number of first kits reported distributed decreased in 2019 and 2020. Since each individual can only pick up a first kit once, it is possible that the population of those interested in carrying naloxone is nearing saturation of first kit collections, whereby those who perceive themselves at risk of an overdose or at risk of witnessing an overdose have previously obtained a kit.

Consistent with increasing THN program shipping volumes to registered sites [29], BC has seen heightened toxicity in the illicit drug supply. Extreme fentanyl concentrations (exceeding 50 µg/L) have been detected in toxic drug-related deaths at higher rates following the public health emergency declaration relating to COVID-19, from 8% between January 2019 and March 2020 (before the declaration) to 14% between April 2020 and May 2021 [7]. The detection rate of benzodiazepines in illicit drug toxicity deaths also greatly escalated in 2020, from 15% in July, to 50% in December [30, 31]. Benzodiazepines can cause respiratory depression that exacerbates opioid overdose [32].

Changes in capacity of harm reduction sites and intermittent closures of some overdose prevention and supervised consumption sites, related to COVID-19, may have motivated people to access naloxone and necessitated increased outreach and peers carrying THN to respond to overdose events [33].

The overwhelming majority (over 91%) of kits reported used were by people who were at risk of overdose i.e. PWUD. In a survey completed by PWUD attending harm reduction supply distribution sites in BC, the vast majority of people who reported administering naloxone when witnessing an overdose had their own THN kit [34]. Since instances of naloxone self-administration are negligible [35], people who are at risk of overdose are using their kits on other PWUD, such as their family, friends, and community members.

We found that a person’s risk of overdose is a significant predictor of having reported using a kit, and the strength of the association depends on their gender. Before stratifying by gender, the pooled odds of someone at risk of overdose having reported kit use was 6.82 times that of someone not at risk of overdose, after adjusting for year, health region, and age. This indicates that most kits are used by PWUD on each other, rather than by bystanders not at risk of an overdose. When analyzing by each gender individually, the odds of women who were at risk of overdose reporting having used a kit were more than 8 times the odds of women who were not at risk of overdose reporting kit use. Such a large observed difference between women at risk and not at risk of overdose for reporting kit use can be a result of a relatively large proportion of women at risk of overdose using their kits, a relatively small proportion of women not at risk of overdose using their kits, or both. We found that for people at risk of overdose, the proportion of women (52.0%) and men (51.4%) that reported kit use was approximately the same. However, among people not at risk of overdose, the proportion of women reporting kit use (11.1%) was noticeably lower than that of men (16.1%). Women also made up the greatest proportion of people collecting first kits (59.5%) (see Additional file 4). This is consistent with the fact that women are more likely to be in caring professions, such as nursing (97%) and social work (83%) [36, 37], potentially resulting in a higher proportion of women with awareness of THN kits who wish to carry them, despite not being at risk of overdose personally. Overall, more research needs to be conducted to evaluate the role of gender in accessing THN training and kits, as well as seeking or providing assistance during an overdose response.

Results pertaining to trans and gender expansive people were unexpected, as previous literature has shown that trans people are more likely to have used illicit drugs associated with a high risk of harm in the past year, compared with their cisgender peers [38, 39]. Factors potentially contributing to the lower odds of reporting having used a kit include experiences of discrimination and stigma during a previous encounter by other THN service users and providers, causing trans and gender expansive people to avoid obtaining replacement kits [40, 41]. Participants may under-report trans and gender expansive status and/or being at risk of overdose so as not to be doubly stigmatized. People who identify as trans and gender expansive also tend to be younger relative to other genders [42].

PWUD continue to be on the frontlines of the toxic drug crisis, and are often the first to witness and respond to overdose events. It is important to recognize the resiliency of communities of PWUD and highlight the fact that members of this population are largely responsible for successes achieved by the THN program to date, in both supporting access to training and kits, as well as saving lives [43]. However, PWUD who respond to overdoses are constantly exposed to loss, trauma, and burnout, which negatively impacts their mental health and wellbeing [44]. While many find a sense of pride and purpose in overdose response [45], it is important to understand the burden placed on communities of PWUD. More social and medical supports are needed for PWUD who face these adversities on a regular basis [44, 46].

Our results indicate that the BC THN program is getting kits into the hands of people who use them most. People who are at risk of overdose represent the majority of people with kits and were found to have much greater odds of using kits compared with people not at risk of overdose across all genders. The odds of someone using a kit has noticeably increased between 2017 to 2020, but we saw that the number of kits reported distributed has increased greatly in 2020. We found that people aged 19–60 had higher odds of reporting used kits compared with individuals that were younger or older, and indeed, the vast majority of kits are being distributed to people in that age range.

Yet, despite expanded access to naloxone in BC, rates of overdose deaths are still rising and more extreme fentanyl concentrations are being seen in toxic drug poisoning deaths. Clearly, naloxone alone is not enough to solve the illicit drug toxicity crisis and additional interventions are necessary as overdose deaths are preventable [47]. An important public health development is BC’s recently announced prescribed safer supply policy, to be implemented over the next three years and designed to address the toxic drug supply crisis [48].

Another approach to reduce toxic drug-related deaths is through decriminalization of illicit drug use and possession. Criminalization of drugs causes stigma against people who use them [49–51]. Negative attitudes against PWUD, including discrimination from law enforcement may cause PWUD to hide their drug use status [52, 53]. In BC, the majority of people using drugs do so alone, increasing the risk of fatal overdose events [54]. Decriminalizing drug use and possession may lead to an increased willingness of PWUD to seek treatment and support services, as well as speak more openly about their drug use with their friends, family, and healthcare providers.

### Strengths and limitations

A strength of this study is the large number of completed distribution records available for analysis (240,606). However, the total number of THN kits shipped in BC between 2017 and 2020 was 841,690 [29]. Thus, distribution records were completed for only 28.6% of all kits that were shipped to THN distribution sites. The authors note that as the number of THN sites and kits shipped as well as the number of overdose events increased, there have been fewer distribution records received, especially from some high-volume sites. Based on correspondence between the BCCDC and a number of high-traffic distribution sites, kits are not being held in reserve and are being continually distributed to community members. It should be noted that this data collection process is intended to be low-barrier and is not a requirement to obtain a kit; additionally, the return of these records is subject to a lag in both the receipt and entry of records.

Some individuals have used kits on multiple, separate occasions. Also, the amount of time that a kit is owned before it becomes used, lost, stolen, confiscated, or expired is not tracked. Thus, it is not possible to know the true incidence or odds of kit use over a certain time interval (for each given year, for example), since it cannot be determined from which year a kit that is reported used was originally distributed.

Responses for certain fields on kit distribution forms, such age, gender, and overdose risk may be asked of kit recipients or assumed by service providers. It must be taken into consideration that stigma surrounding drug use status and gender identity, or fear of identification may cause kit recipients to underreport stigmatized identities, which would introduce bias into the data. In instances where the service provider completes these sections of the reports based on judgement, personal biases may be introduced.

## Conclusions

Between 2017 and 2020, more than 90,000 THN kits were obtained by individuals in BC to replace a kit that was used to reverse an overdose. People at risk of overdose using THN kits on other PWUD account for the overwhelming majority of kits used (and thus, deaths averted) by the BC THN program. While THN has been proven to save lives, additional harm reduction measures are needed to address the illicit drug toxicity crisis in BC and abroad.

## Supplementary Information

**Additional file 1.** 

**Additional file 2.**

**Additional file 3.**

**Additional file 4.**

## Data Availability

All data generated or analysed during this study are included in this published article [and its supplementary information files].

## Abbreviations

AOR: Adjusted odds ratio
AIC: Akaike’s information criteria
BC: British Columbia
BCCDC: British Columbia Centre for Disease Control
CI: Confidence interval
LRT: Likelihood ratio test
PWUD: People who use drugs
THN: Take-Home Naloxone
VIF: Variance inflation factor
