# Supplementary material for: Factors associated with take-home naloxone kit usage in British Columbia: an analysis of administrative data
Source: Subst Abuse Treat Prev Policy. 2022 Mar 31;17:25. doi: 10.1186/s13011-022-00452-8 (PMC8968772; doi:10.1186/s13011-022-00452-8)
Supplement: Supplementary file 1 — Additional file 1. [file 13011_2022_452_MOESM1_ESM.pdf]

TAKE HOME NALOXONE: DISTRIBUTION RECORD

towardtheheart.com — BCCDC HARM REDUCTION SERVICES

Fax/email this form **monthly** to 604.707.2516 or naloxone@bccdc.ca

Site ID# __________ **Full Site Name** ______________________________ **City** ________________

DATE KIT GIVEN OUT	KIT RECIPIENT DESCRIPTION				NOTES **Optional Site Use** (not required by BCCDC)
	OD RISK	GENDER	AGE RANGE (YRS)	1ST KIT OR REPLACEMENT	
MM/DD/YYYY	☐ At risk of OD<br>☐ Not at Risk	☐ Male<br>☐ Female<br>☐ Other	☐ < 19 ☐ 19-30<br>☐ 31-60 ☐ > 60	☐ 1st Kit<br>☐ Replacement (Last Kit Used)<br>☐ Replacement (Other Reason)	
MM/DD/YYYY	☐ At risk of OD<br>☐ Not at Risk	☐ Male<br>☐ Female<br>☐ Other	☐ < 19 ☐ 19-30<br>☐ 31-60 ☐ > 60	☐ 1st Kit<br>☐ Replacement (Last Kit Used)<br>☐ Replacement (Other Reason)	
MM/DD/YYYY	☐ At risk of OD<br>☐ Not at Risk	☐ Male<br>☐ Female<br>☐ Other	☐ < 19 ☐ 19-30<br>☐ 31-60 ☐ > 60	☐ 1st Kit<br>☐ Replacement (Last Kit Used)<br>☐ Replacement (Other Reason)	
MM/DD/YYYY	☐ At risk of OD<br>☐ Not at Risk	☐ Male<br>☐ Female<br>☐ Other	☐ < 19 ☐ 19-30<br>☐ 31-60 ☐ > 60	☐ 1st Kit<br>☐ Replacement (Last Kit Used)<br>☐ Replacement (Other Reason)	
MM/DD/YYYY	☐ At risk of OD<br>☐ Not at Risk	☐ Male<br>☐ Female<br>☐ Other	☐ < 19 ☐ 19-30<br>☐ 31-60 ☐ > 60	☐ 1st Kit<br>☐ Replacement (Last Kit Used)<br>☐ Replacement (Other Reason)	
MM/DD/YYYY	☐ At risk of OD<br>☐ Not at Risk	☐ Male<br>☐ Female<br>☐ Other	☐ < 19 ☐ 19-30<br>☐ 31-60 ☐ > 60	☐ 1st Kit<br>☐ Replacement (Last Kit Used)<br>☐ Replacement (Other Reason)	
MM/DD/YYYY	☐ At risk of OD<br>☐ Not at Risk	☐ Male<br>☐ Female<br>☐ Other	☐ < 19 ☐ 19-30<br>☐ 31-60 ☐ > 60	☐ 1st Kit<br>☐ Replacement (Last Kit Used)<br>☐ Replacement (Other Reason)	
MM/DD/YYYY	☐ At risk of OD<br>☐ Not at Risk	☐ Male<br>☐ Female<br>☐ Other	☐ < 19 ☐ 19-30<br>☐ 31-60 ☐ > 60	☐ 1st Kit<br>☐ Replacement (Last Kit Used)<br>☐ Replacement (Other Reason)	
MM/DD/YYYY	☐ At risk of OD<br>☐ Not at Risk	☐ Male<br>☐ Female<br>☐ Other	☐ < 19 ☐ 19-30<br>☐ 31-60 ☐ > 60	☐ 1st Kit<br>☐ Replacement (Last Kit Used)<br>☐ Replacement (Other Reason)	
MM/DD/YYYY	☐ At risk of OD<br>☐ Not at Risk	☐ Male<br>☐ Female<br>☐ Other	☐ < 19 ☐ 19-30<br>☐ 31-60 ☐ > 60	☐ 1st Kit<br>☐ Replacement (Last Kit Used)<br>☐ Replacement (Other Reason)	
MM/DD/YYYY	☐ At risk of OD<br>☐ Not at Risk	☐ Male<br>☐ Female<br>☐ Other	☐ < 19 ☐ 19-30<br>☐ 31-60 ☐ > 60	☐ 1st Kit<br>☐ Replacement (Last Kit Used)<br>☐ Replacement (Other Reason)	
MM/DD/YYYY	☐ At risk of OD<br>☐ Not at Risk	☐ Male<br>☐ Female<br>☐ Other	☐ < 19 ☐ 19-30<br>☐ 31-60 ☐ > 60	☐ 1st Kit<br>☐ Replacement (Last Kit Used)<br>☐ Replacement (Other Reason)	
MM/DD/YYYY	☐ At risk of OD<br>☐ Not at Risk	☐ Male<br>☐ Female<br>☐ Other	☐ < 19 ☐ 19-30<br>☐ 31-60 ☐ > 60	☐ 1st Kit<br>☐ Replacement (Last Kit Used)<br>☐ Replacement (Other Reason)	
MM/DD/YYYY	☐ At risk of OD<br>☐ Not at Risk	☐ Male<br>☐ Female<br>☐ Other	☐ < 19 ☐ 19-30<br>☐ 31-60 ☐ > 60	☐ 1st Kit<br>☐ Replacement (Last Kit Used)<br>☐ Replacement (Other Reason)	
MM/DD/YYYY	☐ At risk of OD<br>☐ Not at Risk	☐ Male<br>☐ Female<br>☐ Other	☐ < 19 ☐ 19-30<br>☐ 31-60 ☐ > 60	☐ 1st Kit<br>☐ Replacement (Last Kit Used)<br>☐ Replacement (Other Reason)	
