# Supplementary material for: Factors associated with take-home naloxone kit usage in British Columbia: an analysis of administrative data
Source: Subst Abuse Treat Prev Policy. 2022 Mar 31;17:25. doi: 10.1186/s13011-022-00452-8 (PMC8968772; doi:10.1186/s13011-022-00452-8)
Supplement: Supplementary file 2 — Additional file 2. [file 13011_2022_452_MOESM2_ESM.docx]

**Table A2:** **BC THN Program – Reason for kit collection in 2017-2020.**

| **Year** | **Kit Collection Reason** | | | **Total** |
| --- | --- | --- | --- | --- |
|  | **1st Kit** | **Replacement-Other** | **Replacement-Used** |  |
|  | **n (%)** | **n (%)** | **n (%)** | **n** |
| 2017 | 36,886 (58.2%) | 10,938 (17.3%) | 15,523 (24.5%) | 63,347 |
| 2018 | 29,505 (48.6%) | 9,865 (16.3%) | 21,309 (35.1%) | 60,679 |
| 2019 | 23,128 (43.9%) | 8,563 (16.2%) | 21,009 (39.9%) | 52,700 |
| 2020 | 20,887 (32.7%) | 10,823 (16.9%) | 32,170 (50.4%) | 63,880 |
