# Supplementary material for: Factors associated with take-home naloxone kit usage in British Columbia: an analysis of administrative data
Source: Subst Abuse Treat Prev Policy. 2022 Mar 31;17:25. doi: 10.1186/s13011-022-00452-8 (PMC8968772; doi:10.1186/s13011-022-00452-8)
Supplement: Supplementary file 3 — Additional file 3. [file 13011_2022_452_MOESM3_ESM.docx]

**Table A3:** **BC THN Program – Kits reported used by year, stratified by gender**

|  | **Kit Used** | **Kit Not Used** | **Total** |
| --- | --- | --- | --- |
|  | **n (%)** | **n (%)** | **n (%)** |
| **Male** |  |  |  |
| 2017 | 5,315 (30.9%) | 11,884 (69.1%) | 17,199 (17.3%) |
| 2018 | 11,798 (40.5%) | 17,356 (59.5%) | 29,154 (29.2%) |
| 2019 | 10,659 (44.7%) | 13,188 (55.3%) | 23,847 (23.9%) |
| 2020 | 16,781 (56.8%) | 12,757 (43.2%) | 29,538 (29.6%) |
| **Female** |  |  |  |
| 2017 | 3,430 (25.1%) | 10,241 (74.9%) | 13,671 (15.8%) |
| 2018 | 8,162 (32.9%) | 16,683 (67.1%) | 24,845 (28.7%) |
| 2019 | 9,381 (39.4%) | 14,401 (60.6%) | 23,782 (27.4%) |
| 2020 | 12,778 (52.5%) | 11,557 (47.5%) | 24,335 (28.1%) |
| **Trans & Gender Expansive** |  |  |  |
| 2017 | 56 (20.7%) | 215 (79.3%) | 271 (9.7%) |
| 2018 | 207 (24.6%) | 636 (75.4%) | 843 (30.0%) |
| 2019 | 133 (22.6%) | 456 (77.4%) | 589 (21.0%) |
| 2020 | 384 (34.8%) | 720 (65.2%) | 1104 (39.3%) |
