# Supplementary material for: Factors associated with take-home naloxone kit usage in British Columbia: an analysis of administrative data
Source: Subst Abuse Treat Prev Policy. 2022 Mar 31;17:25. doi: 10.1186/s13011-022-00452-8 (PMC8968772; doi:10.1186/s13011-022-00452-8)
Supplement: Supplementary file 4 — Additional file 4. [file 13011_2022_452_MOESM4_ESM.docx]

**Table A4:** **BC THN Program – Reason for kit collection (complete cases) in 2017-2020, stratified by overdose risk of kit recipient, gender and year.**

| **2017** | | | | | |
| --- | --- | --- | --- | --- | --- |
|  | **1st Kit** | **Replacement-Other** | **Replacement-Used** | **Total** |  |
|  | **n (%)** | **n (%)** | **n (%)** | **n (%)** |  |
| **At Risk of OD** |  |  |  |  |  |
| Male | 5,045 (38.3%) | 3,253 (24.7%) | 4,869 (37.0%) | 13,167 (60.9%) |  |
| Female | 3,320 (40.0%) | 1,957 (23.6%) | 3,016 (36.4%) | 8,293 (38.4%) |  |
| Trans & Gender Expansive | 74 (48.0%) | 34 (22.1%) | 46 (29.9%) | 154 (0.7%) |  |
| **Not at Risk of OD** |  |  |  |  |  |
| Male | 3,111 (77.1%) | 475 (11.8%) | 446 (11.1%) | 4,032 (42.3%) |  |
| Female | 4,443 (82.6%) | 521 (9.7%) | 414 (7.7%) | 5,378 (56.5%) |  |
| Trans & Gender Expansive | 83 (71.0%) | 24 (20.5%) | 10 (8.5%) | 117 (1.2%) |  |
| **2018** | | | | | |
|  | **1st Kit** | **Replacement-Other** | **Replacement-Used** | **Total** |  |
| **At Risk of OD** |  |  |  |  |  |
| Male | 7,035 (31.6%) | 4,390 (19.8%) | 10,791 (48.6%) | 22,216 (59.5%) |  |
| Female | 4,596 (31.4%) | 2,799 (19.1%) | 7,233 (49.5%) | 14,628 (39.2%) |  |
| Trans & Gender Expansive | 206 (43.4%) | 113 (23.8%) | 156 (32.8%) | 475 (1.3%) |  |
| **Not at Risk of OD** |  |  |  |  |  |
| Male | 5,060 (72.9%) | 871 (12.6%) | 1,007 (14.5%) | 6,938 (39.6%) |  |
| Female | 8,336 (81.6%) | 952 (9.3%) | 929 (9.1%) | 10,217 (58.3%) |  |
| Trans & Gender Expansive | 222 (60.3%) | 95 (25.8%) | 51 (13.9%) | 368 (2.1%) |  |
| **2019** | | | | | |
|  | **1st Kit** | **Replacement-Other** | **Replacement-Used** | **Total** |  |
| **At Risk of OD** |  |  |  |  |  |
| Male | 5,628 (29.5%) | 3,604 (18.9%) | 9,832 (51.6%) | 19,064 (53.1%) |  |
| Female | 4,892 (29.7%) | 3,077 (18.7%) | 8,504 (51.6%) | 16,473 (45.9%) |  |
| Trans & Gender Expansive | 114 (32.8%) | 125 (35.9%) | 109 (31.3%) | 348 (1.0%) |  |
| **Not at Risk of OD** |  |  |  |  |  |
| Male | 3,354 (70.1%) | 602 (12.6%) | 827 (17.3%) | 4,783 (38.8%) |  |
| Female | 5,689 (77.8%) | 743 (10.2%) | 877 (12.0%) | 7,309 (59.3%) |  |
| Trans & Gender Expansive | 176 (73.0%) | 41 (17.0%) | 24 (10.0%) | 241 (1.9%) |  |
| **2020** | | | | | |
|  | **1st Kit** | **Replacement-Other** | **Replacement-Used** | **Total** |  |
| **At Risk of OD** |  |  |  |  |  |
| Male | 6,266 (23.9%) | 3,950 (15.1%) | 15,995 (61.0%) | 26,211 (56.3%) |  |
| Female | 4,641 (23.7%) | 3,024 (15.4%) | 11,928 (60.9%) | 19,593 (42.1%) |  |
| Trans & Gender Expansive | 282 (39.3%) | 118 (16.4%) | 318 (44.3%) | 718 (1.6%) |  |
| **Not at Risk of OD** |  |  |  |  |  |
| Male | 1,724 (51.8%) | 817 (24.6%) | 786 (23.6%) | 3,327 (39.3%) |  |
| Female | 2,326 (49.1%) | 1,566 (33.0%) | 850 (17.9%) | 4,742 (56.1%) |  |
| Trans & Gender Expansive | 157 (40.7%) | 163 (42.2%) | 66 (17.1%) | 386 (4.6%) |  |
